# Supplementary material for: Identification of photoperiod-induced specific miRNAs in the adrenal glands of Sunite sheep (Ovis aries)
Source: Front Vet Sci. 2022 Jul 22;9:888207. doi: 10.3389/fvets.2022.888207 (PMC9354845; doi:10.3389/fvets.2022.888207)
Supplement: Supplementary file 1 [file Data_Sheet_1.ZIP › Supplementary file/miRDeep2 file.zip!/miRDeep2 file/Novel_Structure/LP42A1/Novel_622.pdf]

Provisional ID	:	NC_019471.2_23212
Score total	:	139.3
Score for star read(s)	:	3.9
Score for read counts	:	131.2
Score for mfe	:	2.6
Score for randfold	:	1.6
Score for cons. seed	:	
Total read count	:	269
Mature read count	:	261
Loop read count	:	0
Star read count	:	8

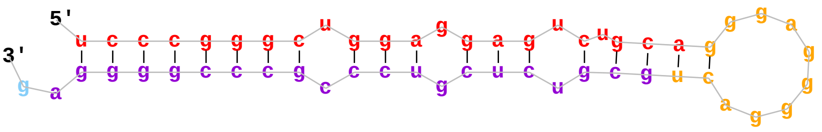

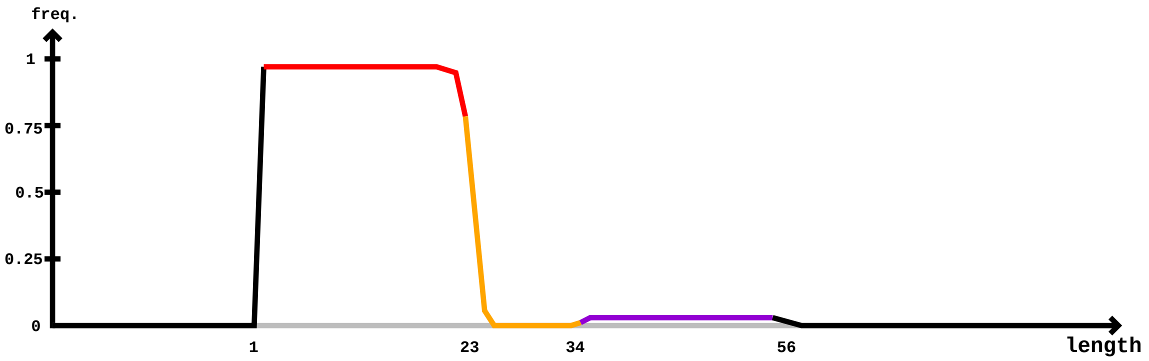

Mature Star

```
5'- ggaccugggcaggggcggggucccgggcuggaggagucugcagggaggggacugcgucucgucccgcccggggagaccgccccuugccgcucccaggacgguggauagc   -3'    obs
    ggaccugggcaggggcggggucccgggcuggaggagucugcagggaggggacugcgucucgucccgcccggggagaccgccccuugccgcucccaggacgguggauagc          exp
    .......(((((((((((..((((((((.(((.(((.(.((((........)))))).))).))).))))))))...))))))).))))(((.(((......)))..)))   reads   mm   sample
    ....................ucccgggcuggaggagucu......................................................................   3       0    seq
    ....................ucccgggcuggaggagucug.....................................................................   3       0    seq
    ....................ucccgggcuggaggagucugc....................................................................   44      0    seq
    ....................ucccgggcuggaggagucugca...................................................................   133     0    seq
    ....................ucccgggcuggaggagucugcag..................................................................   63      0    seq
    ....................ucccgggcuggaggagucugcagg.................................................................   15      0    seq
    .....................................................gcgucucgucccgcccgggga...................................   3       0    seq
    ......................................................cgucucgucccgcccgggga...................................   1       0    seq
    ......................................................cgucucgucccgcccggggag..................................   2       0    seq
    ......................................................cgucucgucccgcccggggaga.................................   2       0    seq
```
